# Chemically and Green Synthesized ZnO Nanoparticles Alter Key Immunological Molecules in Common Carp (*Cyprinus carpio*) Skin Mucus

**DOI:** 10.3390/ijms22063270

**Published:** 2021-03-23

**Authors:** Ghasem Rashidian, Carlo C. Lazado, Heba H. Mahboub, Ramin Mohammadi-Aloucheh, Marko D. Prokić, Hend S. Nada, Caterina Faggio

**Affiliations:** 1 Department of Aquaculture, Faculty of Natural Resources and Marine Sciences, Tarbiat Modares University, Noor 4641776489, Iran; 2 Nofima, Norwegian Institute of Food Fisheries and Aquaculture Research, 1433 Ås, Norway; carlo.lazado@nofima.no; 3 Department of Fish Diseases and Management, Faculty of Veterinary Medicine, Zagazig University, Zagazig 44519, Egypt; hhhmb@yahoo.com; 4 Central Tehran Branch, Department of Biology, Islamic Azad University, Tehran 1469696191, Iran; ramin.p1363@gmail.com; 5 Department of Physiology, Institute for Biological Research “Siniša Stanković”, National Institute of Republic of Serbia, University of Belgrade, 11060 Belgrade, Serbia; marko.prokic@ibiss.bg.ac.rs; 6 Department of Microbiology, Faculty of Veterinary Medicine, Zagazig University, Zagazig 44519, Egypt; hend.saeed@hotmail.com; 7 Department of Chemical, Biological, Pharmaceutical and Environmental Sciences, University of Messina, Viale Ferdinando Stagno d’Alcontres 31, 98166 S Agata-Messina, Italy

**Keywords:** mucosal immunity, nanotoxicology, green synthesis, nanoparticles, common carp, skin mucus

## Abstract

This study was conducted to compare the effects of commercially available (C) and green synthesized (GS) Zinc oxide nanoparticles (ZnO-NPs) on immunological responses of common carp (*Cyprinus carpio*) skin mucus. GS ZnO-NPs were generated using *Thymus pubescent* and characterized by UV–vis diffuse reflectance spectroscopy (DRS), Fourier-transform infrared spectroscopy (FTIR), X-ray powder diffraction (XRD), scanning electron microscope (SEM), and energy-dispersive X-ray spectroscopy (EDX). Fish (*n* = 150) were randomly allocated into five groups in triplicate and received a waterborne concentration of 0% (control), 25%, and 50% of LC50 96 h of commercially available (C1 and C2) and green synthesized ZnO-NPs (GS1 and GS2) for 21 days. Results from XRD displayed ZnO-NPs with 58 nm in size and UV-vis DRS, EDX, and FT-IR analysis showed that some functional groups from plant extract bonded to the surface of NPs. The SEM images showed that ZnO-NPs have conical morphology. Acute toxicity study showed a higher dose of LC50_96h_ for green synthesized ZnO-NPs (78.9 mg.L^−1^) compared to the commercial source (59.95 mg.L^−1^). The highest activity of lysozyme and alternative complement activity (ACH50) were found in control and GS1 groups. A significant decrease in alkaline phosphatase activity (ALP) was found in C1 and C2 groups compared to other treatments. Protease activity (P) was significantly decreased in the C2 group compared to the control and GS groups. Total immunoglobulin (total Ig) content was the highest in the control. In addition, total Ig in the GS1 group was higher than GS2. The exposure to ZnO-NPs lowered total protein content in all experimental groups when compared to control. Present findings revealed lower induced immunosuppressive effects by green synthesized ZnO-NPs on key parameters of fish skin mucus.

## 1. Introduction

Nanotechnology is one of the most advanced technologies that has emerged from the convergence of physics, chemistry, and biology sciences producing various types of materials including nanoparticles (NPs) with at least one dimension below 100 nm [1,2,3,4,5,6]. Metal oxide nanoparticles, as a very important class of NPs, are highly persistent in the environment and food chain [7] making them very dangerous for the environment and aquatic organisms. Engineered metal and metal-oxide NPs are known to impose a wide range of harmful implications (e.g., inducing oxidative stress, DNA damage, mutations, decreasing cell viability, stimulation of apoptosis and necrosis) on living organisms [8,9,10,11]. Besides, NPs can interact with immune compartments resulting in immunosuppression [12,13]. The toxicity of NPs depends on various factors such as surface-to-volume ratio, phase transfer, chemical stability, solubility, and the tendency to mass formation.

Zinc (Zn) is a nutritional trace element essential for living animals, including aquatic animals [14], however, excessive Zn concentrations can be toxic [15,16]. Zinc oxide nanoparticles (ZnO-NPs) are inorganic compounds with a wide range of applications in various industries from cosmetics to wastewater treatment [17,18,19,20,21,22,23,24]. There is an estimation that 3700 tons per year of ZnO-NPs are released into the aquatic environment around the world [25]. However, it is not possible to have an actual estimation due to the uncontrolled use and release of NPs. Since aquatic environments are believed to be the ultimate receiver of these engineered materials, it is, therefore, important to evaluate the impacts of NPs on fish and other aquatic animals [26,27]. Fish can be also exposed to concentrations of NPs that are greater than environmental ones via food and the process of biomagnification through the food chain. Several studies have been dealing with the toxicity risks of engineered NPs to the aquatic organism [9].

The green synthesis of NPs using plant extracts offers an alternative and promising approach in the production of safer and environmentally friendly nanoparticles [28,29]. According to [30], the eco-friendly biosynthesized NPs provides an alternative to the chemically synthesized ones, which should lower the chemical toxicity in the natural environments. Furthermore, a majority of researchers have proposed green routes (using plant extracts) for the synthesis of ZnO nanomaterials [31]. Using plant extracts with high antioxidant content, e.g., polyphenols and sulfated polysaccharides to synthesize metal nanoparticles, is a perfect option to modify NPs’ specific surfaces area (attachment of functional groups to the surface of NPs) and probably reduce their toxicity to living organisms.

A recent study reported that the chemical synthesized silver NPs is 10 times more toxic than the green synthesized nanoparticles for *Artemia nauplii* [32]. Another recent review recommends the potential use of the green synthesized ZnO-NPs as growth promotors, also, to increase resistance against viral infection [28]. Furthermore, ZnO-NPs have been found to exert cytotoxic activity against cancer cells in addition to their promising antimicrobial activity [33]. The information regarding the toxicity of green synthesized NPs on fish skin mucus is scarcer.

Regardless of the exposure route, the interaction of NPs and immune system compartments is inevitable. A study showed that common carp skin mucus actively responds to waterborne exposure of NPs [34]. Some NPs may elicit immune responses at low concentrations suggesting immunostimulatory or immunosuppressive effects of NPs. However, dietary administration of ZnO-NPs [35,36] and even lower doses of ZnO-NPs cause cytotoxicity, oxidative stress, changes in blood biochemical parameters, and tissue damage. Furthermore, it has been reported that Nile tilapia antioxidant defense system is compromised when exposed to ZnO-NPs [37]. Earlier studies revealed the involvement of NPs in evolving oxidative stress by either inhibiting the antioxidant system of cells [38] or by excessing production of reactive oxygen species (ROS) [39] thus, inducing a toxic impact. 

Thus, the present study was conducted to compare the toxicity of the same concentrations of green synthesized ZnO-NPs (58 nm) using *Thymus pubescent* and commercially available ZnO-NPs (35–45 nm) on major immunological parameters of skin mucus of common carp as a model organism. Common carp (*Cyprinus carpio*) is the fourth important aquaculture species with high economic value [40], which has been suggested as an appropriate model for toxicological studies. To the best of our knowledge, this is the first report to compare the toxicological effects of green synthesized and commercial ZnO-NPs on skin mucus immunological responses of common carp.

## 2. Results

### 2.1. Characterization of the Fabricated ZnO-NPs

**Figure 1:**
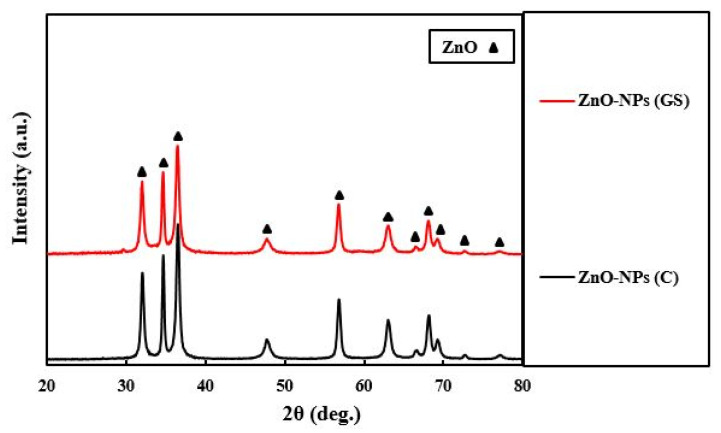
X-ray powder diffraction (XRD) patterns of commercial (C) and green synthesized (GS) ZnO-NPs.

**Figure 2:**
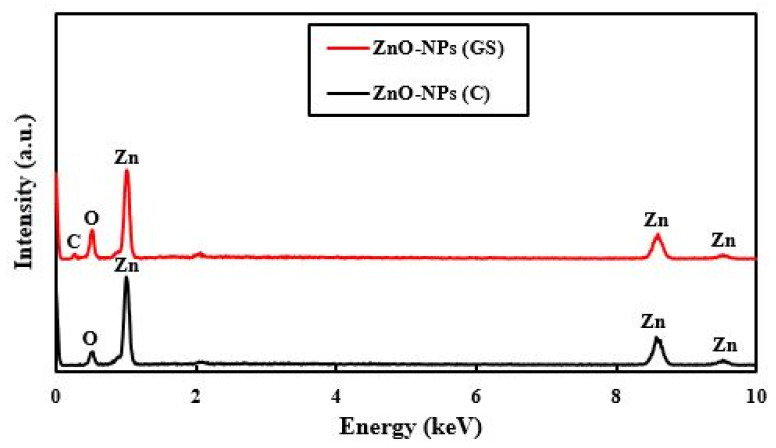
Energy-dispersive X-ray spectroscopy (EDX) spectra of commercial (C) and green synthesized (GS) ZnO-NPs.

**Figure 3:**
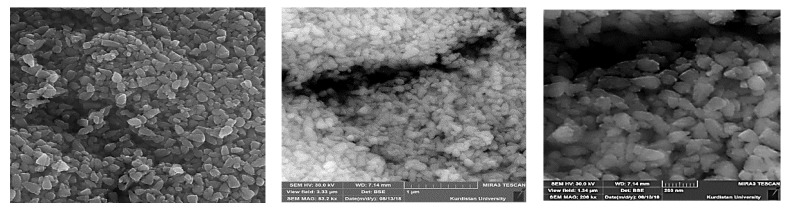
Scanning electron microscope (SEM) image of green synthesized ZnO-NPs.

**Figure 4:**
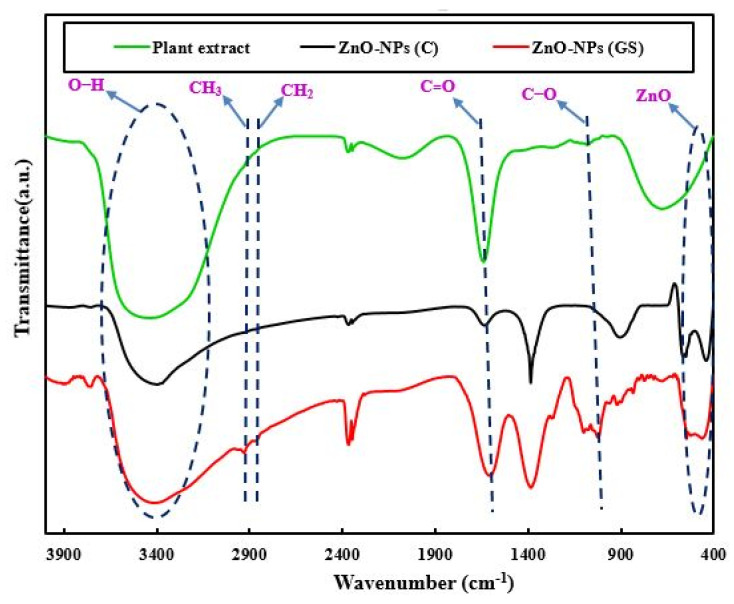
Fourier-transform infrared spectroscopy (FTIR) spectra of *Thymus pubescent* extract, commercial (C) and green synthesized (GS) ZnO-NPs.

**Figure 5:**
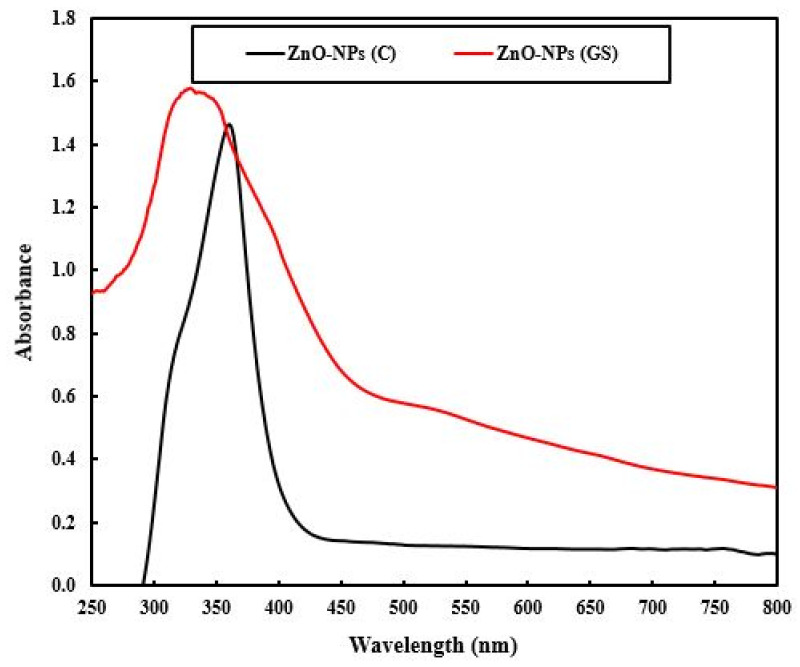
UV–vis diffuse reflectance spectroscopy (DRS) spectra of commercial (C) and green synthesized (GS) ZnO-NPs.

The obtained ZnO-NPs were characterized by XRD, EDX, SEM, FTIR, and UV-vis DRS spectra analysis as shown in Figure 1, Figure 2, Figure 3, Figure 4 and Figure 5. We performed XRD analysis in order to determine the form (amorphous or crystalline) of our samples. Figure 1 represents the results of XRD pattern of prepared ZnO-NPs using *Thymus pubescent*. 

The sharp diffraction peaks confirmed that the obtained ZnO-NPs were crystalline in nature. Scherer’s equation (Equation (1)) was used to calculate the mean crystallite size of the ZnO-NPs.

D = *K*λ/*β*cosθ (1)

where *θ* is Bragg’s angle, *β* is peak width at half maximum, the wavelength of X-ray radiation is λ = 0.15406 nm and *K* is Scherer’s constant, which is 0.9.

Hence, the average crystallite sizes of the ZnO-NPs were determined using Scherer’s equation to be 58 nm. There are no other peaks related to impurities, clearly indicating the high purity of fabricated ZnO-NPs. EDX analysis was exploited to evaluate the chemical composition and purity of the ZnO-NPs, as shown in Figure 2. 

The EDX spectra revealed the presence of zinc, oxygen, and carbon elements. The presence of a carbon element in the EDX was attributed to bonded functional groups of plant origin. In addition, generated NPs were of high purity since no other irrelevant peaks were detected. Furthermore, the weight percentages of zinc (Zn), oxygen (O), and carbon (C) elements were obtained as 78.6, 18.92, and 2.48%, respectively.

Results from SEM analysis are displayed in Figure 3. As can be seen, most of the particles are nearly conical surface structures. 

The observed peak at 466 cm^−1^ in FTIR spectra of ZnO-NPs (Figure 4) was attributed to stretching of the vibration of Zn–O bond. In addition, the peaks at 1073 and 1623 cm^–1^ were attributed to stretching vibrations of the C–O and C=O bonds [41]. The absorption peaks for C–H stretching vibrations of CH_2_ and CH_3_ groups were observed at 2862 and 2934 cm^−1^, respectively [42]. Finally, the FTIR spectra of the extract and all samples showed broad absorption peaks at 3200 to 3668 cm^−1^, representing stretching the vibration of O–H group [43].

UV–vis spectra (Figure 5) showed an absorbance peak at 355 nm which can be described as the intrinsic band-gap absorption of ZnO-NPs due to the electron transitions from the valence band to the conduction band (O_2p_-Zn_3d_). The reduction in the size of ZnO-NPs due to the presence of the extract was observed as a shift of the blue peak of 41 nm. This is attributed to the quantum confinement effect. Furthermore, the ZnO-NPs had strong absorption in the visible region due to the presence of functional groups from the extract.

### 2.2. Toxicity Assessment of ZnO-NPs

**Figure 6:**
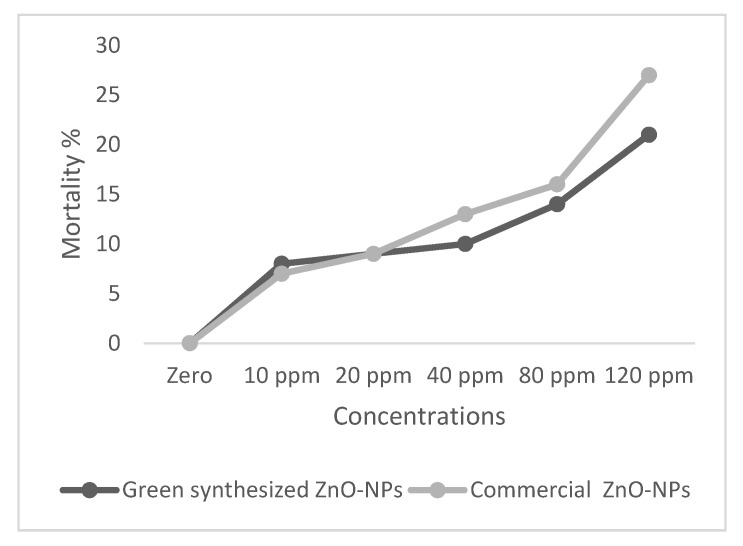
The cumulative mortality of common carp exposed to different levels of commercial and green synthesized ZnO-NPs for 96 h (30 fish for each concentration).

Fish were exposed to different concentrations of commercial and green synthesized ZnO-NPs, mortality was counted for 96 h (Figure 6), and lethal concentrations were calculated accordingly (Table 1 and Table 2). LC50 96 h of commercial and green synthesized ZnO-NPs has recorded 59.95 and 78.9 mg.L^−1^ respectively. 

### 2.3. Skin Mucus Immunological Parameters

Skin mucus immunological responses of common carp exposed to different levels of commercial and green synthesized ZnO-NPs for 21 days are shown in Table 3. Lysozyme activity was statistically highest in the control group, then in the GS1 group in comparison to other treatments. Similar results were observed for alternative complement activity (ACH50), whereas control and GS1 groups had higher activities than other groups. As for alkaline phosphatase, groups treated with commercial ZnO-NPs showed statistically lower activity in comparison to control groups. Among ZnO treated groups, the lowest activity of alkaline phosphatase was observed in the C1 group (22.89 ± 0.40) and the highest in GS1 (24.96 ± 0.61). Protease activity was significantly decreased in fish exposed to ZnO-NPs except for a lower concentration of green synthesized ZnO-NPS (GS1), which did not show the statistical difference when compared to the control group. Total Ig was found the highest in the control group followed by a lower concentration of green synthesized NPs (GS1) compared to other treatments while no significant difference was found among GS2, C1, and C2 treatments. Similar to the results of total Ig, exposure to ZnO-NPs induced a decrease in the total protein content where the highest value of total protein was measured in the control group. Differences between ZnO-NPs treated revealed that total protein was higher in individuals from a lower concentration of green synthesized NPs (GS1) group.

**Figure 7:**
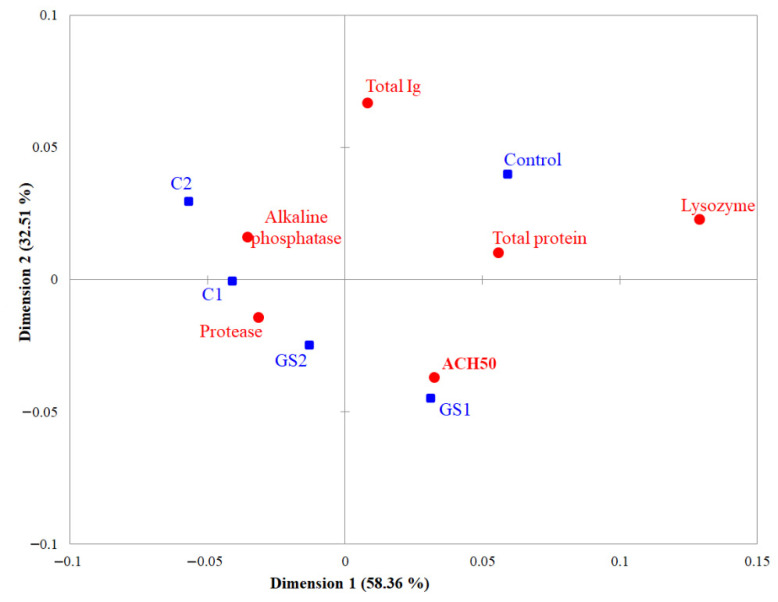
Correspondence analysis ordination plot and position of the five analyzed groups (control, GS1, GS2, C1, and C2) to the analyzed immune parameters.

Correspondence analysis showed that the first two dimensions explain 90.87% of total inertia (Figure 7). Control and GS1 groups were clearly separated from other groups by first dimension (58.37% of inertia). The second dimension (32.51%) separated the control and C2 groups from GS1 and GS2 groups, while the C2 was placed on the axis. The parameters distinguished examined groups were for: control individual- total Ig, total protein and lysozyme; GS1- complement; C1 and GS2- protease; and C2- alkaline phosphatase.

## 3. Discussion

Nanotechnology has gained tremendous attention to enhance aquaculture with advanced Nano-tools. There is a common understanding that the final destination of released NPs would be the environment, underground or open water reservoirs. It is very likely that NPs enter the fish body via food intake and ingestion of rearing water, however, several other possible routes are suggested, e.g., epithelium of gills and skin [36,44,45]. In addition, the toxicity level of NPs has been shown to depend on various factors including size and shape, a period of exposure, concentration, aggregation, surface area and charge. Herein we report that based on our findings, the green synthesized type of nanoparticles (ZnO-NPs) exhibited relatively less immunotoxic effects on fish skin mucus immunological parameters and probably could be a better choice in comparison to the chemically synthesized type.

The state of immunosuppression in fish living in an aquatic ecosystem can be a possible opportunity for skin inhabiting microorganisms to exert their action [46,47,48]. 

The fish skin is covered with a mucosal surface, as the outer layer, that is always in contact with the surrounding environment. It serves as a major immunological barrier and is considered the first line of defense against pathogens and other imminent stressors [49,50,51]. In addition, mucus, the glycopolymers secretion covering the mucosa, harbors a variety of antibacterial components, including proteins and enzymes, such as lysozyme and proteolytic enzymes, immunoglobulins, complement proteins, lectins, and C-reactive proteins [52,53,54]. Several literature data have addressed the ZnO-NPs toxicity following waterborne exposure to various aquatic organisms [55,56,57]. The innate immune parameters including mucus analysis of the fish skin, leukocytes function, NPs internalization, cytokine expression, and lysozyme level are promising biomarkers to assess fish health upon exposure to NPs [58].

In general, it is suggested that positively charged NPs might bind to mucoproteins and therefore trap within the layer reducing their penetration onto the body and circulation system. This entrapment normally results in the secretion of large amounts of superficial mucus and reduces the chances of NPs passing through ion exchange channels. However, a different interpretation discussed by [59] suggests the strong action of NPs to cross biological barriers and exert different toxic actions, due to the generation of reactive oxygen species (ROS).

To date, three studies demonstrated the effect of NPs exposure on fish skin mucus. Oliveira et al. [60] showed the sensitivity of the skin mucus *Sparus aurata* to a low concentration of gold NPs by measuring the total antioxidant capacity and esterase activity.

Hawkins et al., [61] monitored the amount of mucus production and recorded an increase in mucus production in *Pimephales promelas* gills after 4 h exposure to silver nitrate NPs (AgNO_3_) that persisted until 24 h. Then, on the third day, the amount of mucous production showed a remarkable decrease. The third study displayed the significant impact of NPs on several innate immune parameters, displaying profound alterations in skin mucus, the function of leukocytes (macrophages and neutrophils), NPs internalization, expression of cytokine, and lysozyme level [58].

Recent research recommends the safe use of green synthesized nanoparticles as they contain many bioactive molecules such as polyphenols, enzymes, esters, polysaccharides, and terpenoids). In addition, plant leaf extracts could act as stabilizing and reducing agents in the biosynthesis processes of nanoparticles [33,62].

Fish mucus is enriched with many immune-associated proteins such as lysozymes, phosphatases, immunoglobulins and proteins [63,64,65]. Lysozyme is a potent defense component of the innate immune system through its antibacterial action [66]. Mucus metabolites, including protease activity, are considered good non-invasive bioindicators to determine fish immunological and physiological responses, the composition of these metabolites responded to the many challenges, for example, mucus protein decreased significantly following pathogenic bacterial infection in sea bass [67]. Herein we report the highest level of lysozyme activity in the control and the GS1 groups compared to other groups. It is probable that the green synthesized ZnO-NPs contain a potential substrate for lysozyme thus the induction of lysozyme activity. However, a different perspective mentioned by [58], stated that NPs cause suppression of the fish lysozyme activity. Lysozyme activity was decreased in fish exposed to waterborne concentrations of commercial and green synthesized ZnO-NPs. In contrast, other studies reported that dietary administration of low concentrations of ZnO-NPs elevates lysozyme activity. Until now, few studies evaluated the activity of lysozyme in fish mucus after exposure of fish to NPs. Nevertheless, *Epinephelus coioides* exposure to Cu-NPs and copper sulfate NPs (CuSO_4_) showed suppression in the lysozyme activity in the intestine after 25 days [68]. This diminishing impact was also found in blood samples after 60 days of exposing *Oreochromis niloticus* to Fe_2_O_3_ NPs [69]. Another study revealed a decrease in the serum lysozyme activity after exposure to a low concentration of ZnO-NPs for 2 weeks [70].

Immunoglobulins play a substantial role in the local adaptive immune responses of fish mucous by defending the mucosa against different infections and interact with microorganisms to sustain the commensal homeostasis [71]. The current investigation demonstrated that the green synthesized ZnO-NPs especially at low concentrations have an immunomodulatory activity as shown by the elevation of the total Ig, protease activity, ALP, and total protein compared to the groups administered with the commercial NPs. This result corroborated recent studies demonstrating that the commercial type of ZnO-NPs shows higher toxic effects likely because of their ability to release Zn ions, and cause cell apoptosis [72,73]. Moreover, a comparison of commercial and green synthesized ZnO-NPs has revealed the efficiency of the green synthesized NPs [74] which could be attributed to the presence of bioactive molecules [33,62]. Similar findings were recorded for the toxicity of commercial ZnO-NPs that significantly decreased ALP in common carp post-exposure [75]. In addition, a significant alteration in the total protein and protease of *M. rosenbergii* post-exposure to 90 days of ZnO-NPs were reported [76].

Various skin mucus proteins are targets to identify the fish physiological status and classified into three main groups based on their functions including structural (actins, keratins, and their catabolic products), metabolic (glycolytic enzymes and proteasome-associated proteins), and protection-related group (heat shock proteins, hemopexin, and transferrin) [77]. Proteases are another factor in fish mucus. Proteins such as trypsin, cathepsin BL, cysteine protease (cathepsin D), aspartic protease (and metalloproteases) have been identified in the mucus of fish skin. Antioxidant power activity in the skin mucus owing to the presence of protease blocks bacterial growth [78].

## 4. Materials and Methods

All chemicals used in this experiment were of analytical grade and purchased from local suppliers. Analytical grade compounds of zinc (II) nitrate [Zn (NO3)_2_.6H_2_O], and sodium hydroxide were obtained from Merck. Commercial ZnO-NPs (US3580, NanoSany, Iran) (35–45 nm, 99% purity, spherical shape) were purchased from NanoSany, Iran. The fresh plant materials were obtained from local traders (Ardabil, Iran) and further identified by a botanist.

### 4.1. Preparation of the Extract and Nanoparticles

**Figure 8:**
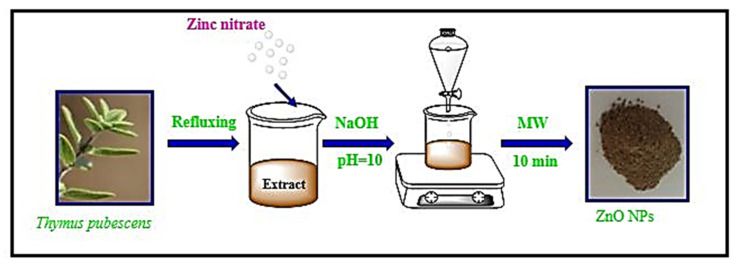
Fabrication scheme of ZnO-NPs from Zinc nitrate using leaf extract of plant *Thymus pubescens.*

The green leaves of *Thymus pubescens* were washed with distilled water and dried at 25 °C for 6 days. Then, 100 mL of distilled water was added to the powdered dried materials (20 g) and the resulting solution was incubated at 95 °C for 24 h and thoroughly mixed using a magnetic starrier (180 rpm) to allow a proper extraction. Finally, the solution was centrifuged at 5000× *g* for 5 min and kept at 4 °C until use. For the preparation of the ZnO-NPs, zinc nitrate (5 g) was dissolved in 80 mL distilled water and 20 mL of the extract at 37 °C under stirring. Afterward, 5 M NaOH was added to the solution in a drop-wise manner until pH 10 was achieved. The suspension was then placed in a microwave oven for 10 min. The precipitate was centrifuged at 2000× *g* for 6 min, washed with distilled water and ethyl alcohol, and dried at 60 °C overnight (Figure 8). 

### 4.2. Characterization of Fabricated NPs

A microwave oven (Panasonic, 2.45 GHz, and 1000 W) was used for the preparation of the samples. FTIR spectra were recorded with KBr pellets using a WQF-510 FTIR Rayleigh. UV-Vis spectra were recorded with T80 double beam UV-Vis spectrophotometer (NORDANTEC-GmbH, Germany) diode-array spectrometer using quartz cells of 1 cm optical path. X-ray diffractometer (XRD) was applied to determine purity, crystallinity, and the average size of NPs. Scanning electron microscopy (SEM) (LEO1450 VP, Germany) with a scanning range from zero to 30 keV was used to assess the shape and morphology of NPs. The purity of the materials by EDX spectroscopy using the same SEM instrument was obtained.

### 4.3. Assessment of LC50 of ZnO-NPs

In order to determine the LC50 of commercial and green-synthesized available ZnO-NPs, 180 fish with an average body weight of 25.41 ± 0.87 g were exposed to 6 different concentrations of 0 (as control), 10, 20, 40, 80, and 120 mg.L^−1^ for 24, 48, 72, and 96 h. Mortalities were counted and LC50 96 h was defined as the concentration of ZnO-NPs capable of killing 50% of fish after 96 h.

### 4.4. Fish Husbandry

Common carp fingerlings were provided from a local farm and transferred to the laboratory (Private facility, Karaj, Iran). Fish were allowed to adopt laboratory conditions for two weeks. The experiment was performed in a completely randomized design with five treatment groups in triplicate. Thus, 150 fish (29.59 ± 0.61 g) were distributed in 15 tanks and fed on commercial carp feed with the approximate composition of 36% protein, 6% lipid, 10% moisture, 11% ash, 5% fiber, and 1.5% phosphorus (Faradaneh.Co, Iran). Fish were fed 2% of their body weight twice a day and uneaten food was siphoned from the bottom of tanks just before the water exchange process. Physiochemical parameters of rearing water including temperature, pH, dissolved oxygen, and hardness (CaCO_3_) were measured as 25± 1 °C, 7.25 ± 0.34, 6.5 ± 0.6 mg.L^−1^, and 385 ± 51 respectively. The photoperiod was set as 12 L:12 D using artificial light. Dead fish were immediately removed and recorded if there was any during the 21-day experiment.

### 4.5. Exposure Experiment

Fish were exposed to waterborne concentrations of 0%, 25%, and 50% of the LC_50_ 96 h (60 ppm) of commercially available (C) or green synthesized (GS) ZnO-NPs referred to as control, C1 (25%), C2 (50%), GS1 (25%), and GS2 (50%) respectively were provided in a total water volume of 200 L for 21 days. Water was exchanged with 30% of freshwater with the same concentrations of NPs to maintain water quality and refresh the NPs concentration. The tanks were vigorously aerated to keep the particles most suspended possible. 

### 4.6. Sampling Process

At the end of the experiment, nine fish were randomly taken from each treatment and sampled for their skin mucus. Fish were starved for 24 h prior to sampling then anesthetized with clove powder (50 ppm) for 5 min. Mucus samples were collected according to [79] with slight modifications. In brief, fish were placed in a small plastic bag containing 50 mL of physiological serum and kept for 1 min. The fish body was gently rubbed and the released mucus was collected. Samples from each three fish from the same replicate were pooled in order to reduce the individual influence and have enough samples for further investigation. Collected samples were centrifuged at 10,000× *g* for 5 min and the supernatant was kept at −80 °C until further analysis.

### 4.7. Skin Mucus Immunological Parameters

At the end of the 21-day exposure experiment, the following mucosal parameters of lysozyme, total immunoglobulin (total Ig), complement activity (ACH50), protease activity (P), alkaline phosphatase (ALP) activity, and total protein (TP) were evaluated. 

#### 4.7.1. Lysozyme Activity

Lysozyme activity was determined according to [80] method. In brief, aliquots of *Micrococcus luteus* were mixed with mucus samples and optical density (OD) was measured using a spectrophotometer (Eppendorf, Germany) at 670 nm. One unit of lysozyme activity was defined causing a reduction in absorbance at 0.001/min.

#### 4.7.2. Total Immunoglobulin (total Ig)

Total Ig was determined following the method described by [81] that is subtracting the total protein of serum before and after treating with 12% polyethylene glycol (PEG, Sigma).

#### 4.7.3. Alternative Complement Activity (ACH50)

Alternative complement activity (ACH50) was measured based on [82]. In brief, sheep red blood cells and gelatin veronal buffer (Sigma, USA) were applied. Each 20-μL aliquots of a serially diluted serum with ethylene glycol-bis(β-aminoethyl ether)-N,N,N′,N′-tetraacetic acid -Mg2+-gelatin veronal buffer (GVB) as a complement source was introduced into 6 μL of sheep red blood cells (SRBC)suspension (4 × 108 cells ml^−1^), and later they were incubated at 21 °C and pH 7.2 for 2 h. Two hundred μL of GVB containing 10 mM Ethylenediaminetetraacetic acid (EDTA) was added into the suspension to stop the hemolytic activity. The suspensions were centrifuged at 1600× *g* for 10 min at 4 °C and OD was read at 414 nm using an ELISA microplate reader. The reactions were supplemented with 6 μL EDTA-GVB, 20 μL EDTA-GVB, and 220 μL distilled water to replace the SRBC suspension, the diluted mucus sample, and the diluted mucus + EDTA-GVB, respectively, as the SRBC blank, serum blank, and 100% hemolysis sample. The ACH50 activity level of hemolysis was calculated for all treatments.

#### 4.7.4. Protease Activity

Mucosal protease activity was measured by the azocasein hydrolysis method described by [83] using commercially available Pars Azmun kits (Tehran, Iran).

#### 4.7.5. Alkaline Phosphatase Activity

In order to measure skin mucus alkaline phosphatase activity (ALP), 1 unit of samples was diluted in 9 unit physiological serum. ALP activity was measured using commercial kits (ALP 5018, Pars Azmun. Co, Tehran, Iran) through the spectrophotometric method following the manufacturer’s instructions.

#### 4.7.6. Total Protein

Total protein levels were determined using commercial kits (Pars Azmun Co., Tehran, Iran) according to the manufacturer’s instructions. The spectrophotometer device was calibrated using provided reagents and after 5 min incubation in ambient temperature total protein content of mucus, samples were measured at 546 nm.

### 4.8. Statistical Analysis

Outliers were checked by Grubb’s test, while normality and homogeneity of variances, Kolmogorov-Smirnov test Levine’s test respectively. Possible differences between control and treatments were analyzed using one-way ANOVA. The post hoc Tukey HSD (honest significant difference) was performed to determine further differences for each variable. The significance level was set as *p* < 0.05. All statistical analyses were performed using STATISTICA 8.0 (StatSoft, Inc., Tulsa, OK, USA, 2007). Correspondence analysis was applied to obtain the position of treatments in the relation to examined parameters using XLSTAT software Version 2015.5.01.22537 and Microsoft Excel 2010.

## 5. Conclusions

Our study suggests the replacement of commercial ZnO-NPs with natural plants synthesized ZnO-NPs as less toxic, and more ecofriendly. To date, this is the first report describing the effects of green synthesized ZnO-NPs on the mucosal immunological parameters of common carp compared with the commercial NPs. In view of current results, future studies are necessary to assess more characteristics of the green synthesized ZnO-NPs in various applications in aquaculture and on different fish species. In addition, methods to ensure the safety, mitigate adverse impacts of ZnO-NPs, and assess their potentially hazardous effects on human health are warranted.

## Figures and Tables

**Table 1:** Lethal concentrations of commercial ZnO-NPs at different time points of 24, 48, 72, and 96 h.

	Concentration (ppm)			
Point	24 h	48 h	72 h	96 h
LC30	230.36	125.95	73.08	32.1
LC50	312.51	184.51	117.62	59.95
LC70	394.66	243.08	162.16	87.49
LC90	513.27	327.64	226.47	127.26
LC99	676.94	444.33	315.22	182.14

**Table 2:** Lethal concentrations of green synthesized ZnO-NPs at different time points of 24, 48, 72, and 96 h.

	Concentration (ppm)			
Point	24 h	48 h	72 h	96 h
LC30	145.76	92.46	43.9	39.31
LC50	179.51	161.43	87.84	78.9
LC70	213.26	230.4	131.87	118.49
LC90	261.99	329.98	195.22	175
LC99	329.23	467.39	282.77	254.52

**Table 3:** Skin mucus immunological parameters (lysozyme, alternative complement activity (ACH50), alkaline phosphatase, protease activity, total Ig and total protein) of common carp exposed to waterborne concentrations of 0% (control), 25% and 50% of the LC_50_ 96 h (60 ppm) of commercially available ZnO-NPs (C1 and C2), and same concentrations of green synthesized ZnO-NPs (GS1 and GS2) for 21 days.

Treatment	Control	GS1	GS2	C1	C2
Lysozyme (U mL^−1^)	9.89 ± 0.37 ^a^	8.07 ± 0.21 ^b^	6.71 ± 0.38 ^c^	5.83 ± 0.21 ^cd^	5.4 ± 0.18 ^d^
ACH50 (U mL^−1^)	32.88 ± 1.31 ^a^	33.64 ± 1.32 ^a^	27.93 ± 0.87 ^b^	25.69 ± 0.33 ^bc^	24.11 ± 0.70 ^c^
Alkaline phosphatase (U L^−1^)	27.61 ± 0.70 ^a^	24.96 ± 0.61 ^b^	24.85 ± 0.57 ^b,c^	22.89 ± 0.40 ^c^	23.60 ± 0.85 ^b,c^
Protease (U mL^−1^)	42.58 ± 1.58 ^a^	42.84 ± 1.41 ^a^	38.04 ± 0.77 ^b^	38.38 ± 1.00 ^b^	36.21 ± 1.04 ^b^
Total Ig (mg mL^−1^)	19.33 ± 0.37 ^a^	15.67 ± 0.37 ^b^	13.47 ± 0.32 ^c^	14.47 ± 0.40 ^c,d^	14.70 ± 0.29 ^b,d^
Total protein (mg mL^−1^)	3.04 ± 0.08 ^a^	2.64 ± 0.07 ^b^	2.35 ± 0.07 ^c^	2.13 ± 0.04 ^d^	2.01 ± 0.06 ^d^

Different letters indicate significant differences between treatments (*p* < 0.05). Data represent mean ± SE.

## Data Availability

The data presented in this study are available on request from the corresponding author. The data are not publicly available due to being a part of a bigger and undergoing project.
